# First-in-Human Phase 0 Study of AB001, a Prostate-Specific Membrane Antigen–Targeted ^212^Pb Radioligand, in Patients with Metastatic Castration-Resistant Prostate Cancer

**DOI:** 10.2967/jnumed.124.269299

**Published:** 2025-05

**Authors:** Kjetil Berner, Eivor Hernes, Monika Kvassheim, Mona-Elisabeth Revheim, Julie Bastiansen, Silje Selboe, Charlotte L. Bakken, Simen R. Grønningsæter, Øyvind S. Bruland, Roy H. Larsen, Lily Bouzelmat, Vicki L. Jardine, Caroline Stokke

**Affiliations:** 1 Department of Oncology, Oslo University Hospital, Oslo, Norway;; 2 Department of Nuclear Medicine, Division of Radiology and Nuclear Medicine, Oslo University Hospital, Oslo, Norway;; 3 Department of Physics and Computational Radiology, Division of Radiology and Nuclear Medicine, Oslo University Hospital, Oslo, Norway;; 4 Institute of Clinical Medicine, University of Oslo, Oslo, Norway;; 5 Intervention Centre, Oslo University Hospital, Oslo, Norway;; 6 Department of Physics, University of Oslo, Oslo, Norway;; 7 Sciencons AS, Oslo, Norway; and; 8 ARTBIO Limited, London, United Kingdom

**Keywords:** mCRPC, PSMA, ^212^Pb, imaging, α-radioligand therapy, targeted α-therapy

## Abstract

AB001, a prostate-specific membrane antigen (PSMA)–targeted small molecule labeled with the in vivo–generating α-emitter ^212^Pb, was investigated in a phase 0 trial in patients with metastatic castration-resistant prostate cancer (mCRPC). The primary objective was to explore the feasibility of γ-camera imaging to assess biodistribution and uptake in metastatic lesions. **Methods:** Three patients with progressive mCRPC and Eastern Cooperative Oncology Group performance status 1 were included, having prostate-specific antigen levels of 0.44, 0.75, and 15 µg/L. All had at least 3 PSMA-expressing metastatic lesions, with an SUV_max_ range of 10.1–77.4 on PSMA PET. Each patient received a microdose of 9.4 ± 0.3 MBq of AB001 intravenously. Planar γ-camera and SPECT/CT imaging was scheduled 1–3 h and 16–24 h after administration. Whole-body clearance was assessed with NaI probe measurements. Activity of ^212^Pb in whole blood and plasma was measured to investigate clearance from blood and in vivo stability of the ligand. Safety, tolerability, and efficacy biomarkers (prostate-specific antigen, alkaline phosphatase) were followed for 28 d. **Results:** AB001 uptake in the lesion with the highest PSMA expression, a retrocaval lymph node metastasis with a short-axis diameter of 11 mm, was visualized on SPECT. Uptake of AB001 was not clearly demonstrated for other metastatic lesions, possibly because of the lower PSMA expression of these metastases on PSMA PET, combined with the administered AB001 microdose and imaging system limitations. Kidney, urinary bladder with contents, and liver uptake of AB001 were clearly distinguishable from adjacent tissue, and the blood pool content was seen. Salivary glands were not visualized. Blood analyses indicated stability of AB001 after injection, and whole-body probe measurements demonstrated an effective half-life of 8 h. There were no complications related to injection of AB001 or adverse reactions during follow-up. As expected for a phase 0 study, there was no indication of therapeutic effects as assessed by prostate-specific antigen and alkaline phosphatase. **Conclusion:** The ^212^Pb-based radioligand AB001 was safely administered to mCRPC patients. γ-camera imaging of AB001 was feasible, even at a microdose, and demonstrated metastatic targeting, albeit for only 1 lesion. The promising biodistribution and clearance encourage further clinical investigation.

## Visual Abstract

**Figure:**
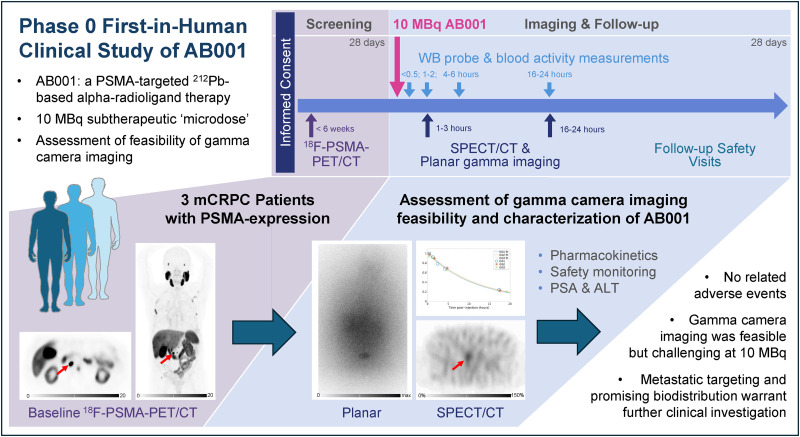

Patients with metastatic castration-resistant prostate cancer (mCRPC) still have unmet treatment needs and a poor prognosis on progressive disease (1). Prostate-specific membrane antigen (PSMA) is commonly overexpressed in prostate cancer, with expression increasing in advanced mCRPC, and limited expression in extraprostatic tissues. Exploiting these properties, PSMA-targeted theranostics have emerged and demonstrated promising clinical results (1). The β-emitting therapy [^177^Lu]Lu-PSMA-617 (^177^Lu-vipivotide tetraxetan; Pluvicto [Novartis]) has demonstrated improved overall survival and radiographic progression-free survival in advanced mCRPC, yet 30%–50% of patients do not respond to treatment (2). Interest in α-radioligand therapy has increased in recent years (3,4), with early clinical data for PSMA-targeted α-radioligand therapies indicating promising anticancer activity in mCRPC, importantly including some cases unresponsive to β-emitting treatments (5–7).

^225^Ac-PSMA–targeted treatments are the most extensively studied α-radioligand therapies; however, ^213^Bi-PSMA has also been studied clinically (8,9). ^212^Pb is an in vivo α-particle generator, with physical decay properties fitting well with the rapid tumor uptake achievable from small-molecule or peptide-targeting agents. Furthermore, ^212^Pb is a generator-based radionuclide suitable for large-scale production (10). In preclinical studies, a new [^212^Pb]Pb-PSMA–targeting ligand, AB001 (formerly ^212^Pb-NG001), showed good tumor uptake, inhibited tumor growth, and had lower kidney uptake than ^212^Pb-labeled PSMA-617 (11,12).

Direct imaging of treatments relying on α-emissions is challenging because of the relatively small amounts of activity used, most often a low intensity of sparse γ-emissions, and sometimes down-scatter from high-energy γ-emissions (13). ^212^Pb decays to the α-particle emitter ^212^Bi via β-particle emission with a half-life of 10.64 h (Fig. 1). A γ-emission of 238.6 keV with an intensity of 43.6% and x-rays (74–90 keV) with a total intensity of 33.5% are emitted in the decay to ^212^Bi (14). Initial clinical imaging of a therapeutic amount of activity of other ^212^Pb-based therapies has been demonstrated in a small study using planar scintigraphy and in 2 case studies using SPECT imaging (15–17).

**FIGURE 1.**
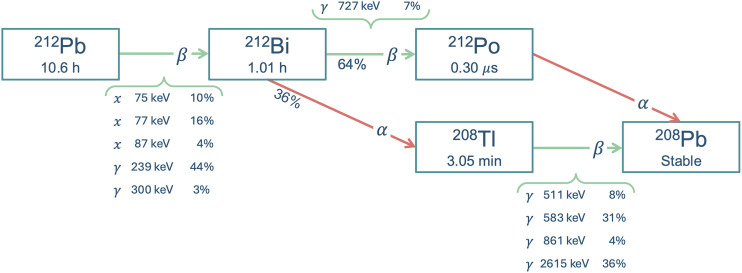
Decay scheme for ^212^Pb, including photon emissions with energies higher than 70 keV and intensities larger than 3% (all photon intensities given per ^212^Pb decay). ^212^Pb emits 1 α-particle per decay via its daughter ^212^Bi. Most imageable photons are emitted in decay to ^212^Bi. High-energy emission of 2.6 MeV from ^208^Tl has intensity of 36% per ^212^Pb decay and causes significant scatter that can degrade ^212^Pb images. (Data reference: NuDat [National Nuclear Data Center, Brookhaven National Laboratory, United States].)

This phase 0 first-in-human study of AB001 in patients with mCRPC was designed to explore the feasibility of γ-camera imaging after a 10-MBq microdose, investigate biodistribution and metastatic lesion uptake, and evaluate assessed safety, activity, and clearance.

## MATERIALS AND METHODS

### Study Design

This was a single-site, open-label, nonrandomized, noncontrolled interventional first-in-human phase 0 study conducted at Oslo University Hospital. Eligible patients were at least 18 y old with progressive mCRPC on standard-of-care treatment and PSMA-expressing mCRPC lesions confirmed by [^18^F]F-PSMA PET/CT. Other inclusion criteria included an Eastern Cooperative Oncology Group performance status of 0–2 (18), life expectancy of more than 6 mo, pathologic confirmation of prostate carcinoma, and adequate organ and bone marrow function. Exclusion criteria included concurrent cancer diagnosis or another cancer diagnosis within the last 2 y, concomitant diseases not compatible with radionuclide therapy, and prior PSMA-targeted radioligand therapy.

Baseline assessment included demographics, medical history, laboratory tests, and physical examination. Safety, tolerability, and efficacy biomarkers (prostate-specific antigen, alkaline phosphatase) were followed up for 28 d after administration of AB001. Common Terminology Criteria for Adverse Events, version 5.0, were used to grade treatment-emergent adverse events. The study was approved by the institutional review board, the Regional Ethical Committee, and the Norwegian Medical Products Agency. Eligible patients gave written informed consent before inclusion in the study.

### Investigational Medicinal Product

AB001 is a small-molecule PSMA-targeting radioligand (previously referenced as ^212^Pb-NG001) using a tetra-carbamoylmethyl cyclododecane chelator to conjugate ^212^Pb (11,12). ^212^Pb was obtained from a ^228^Th/^212^Pb radionuclide generator based on the radon gas diffusion principle (19) at more than 99.99% purity as measured by high-purity germanium detector. A ^212^PbCl_2_ solution at 15–20 MBq/mL was transferred to a single radiolabeling vial, pH was adjusted using sodium acetate, and AB001 ligand was added before mixing at room temperature for 10–15 min. The resultant radiolabeled AB001 was dissolved in 0.9% NaCl with a 5 mg/mL solution of human serum albumin buffered with sodium acetate to a pH of 4.5–6.5. The final investigational medicinal product comprised 1.5–1.7 MBq of ^212^Pb and 2.0 µg of AB001 ligand per milliliter. Between 5.5 and 6.6 mL were used for intravenous injection; 9.4 ± 0.3 MBq of AB001 (9.6, 9.5, and 9.0 MBq for patients ID01, ID02, and ID03, respectively) and a maximum 13 µg of ligand were administered.

### PSMA PET/CT

[^18^F]F-PSMA PET/CT was performed at baseline to assess the presence of PSMA-expressing mCRPC lesions, defined by an image signal at least 3 times that of adjacent normal tissue. Each patient had a [^18^F]F-PSMA PET/CT scan within 5 wk of investigational medicinal product administration performed on a GE HealthCare Discovery MI (patient ID01) or a GE Healthcare Discovery 690 (patients ID02 and ID03) PET/CT scanner. A routine protocol was applied, with patients fasting for approximately 4 h before intravenous injection of 200–252 MBq of ^18^F-PSMA-1007 and image acquisition from the vertex to the upper thigh about 2 h after injection (20). QClear (GE HealthCare) with β-factor 500 was used for PET image reconstructions. SUV_max_ and SUV_mean_ were assessed by isocontouring of lesions at 40% of SUV_max_ or by volumes of interest for normal tissue using a Siemens SyngoVia workstation (version VB60A_HF01).

### γ-Camera Imaging

γ-camera imaging was performed approximately 2 h (day 0) and 18–20 h (day 1) after AB001 injection on a Siemens Symbia Intevo Bold SPECT/CT device with high-energy collimators. For both planar images and SPECT images, 2 energy windows were acquired simultaneously, a 40% window centered on 79 keV and a 20% window centered on 239 keV, with dual scatter windows of 20% and 5%, respectively. The planar images were acquired with a scan speed of 0.5–1.2 mm/s on both days, and the 2 energy windows were summed. The SPECT images were acquired for 20–40 min per bed position, with body contouring orbits, a 256 × 256 matrix, and 60 views, with acquisition during steps. The SPECT images were reconstructed with Flash3D (AutoRecon), with 30 iterations and 2 subsets for day 0 images and 30 iterations and 1 subset for day 1 images. Triple-energy-window scatter correction, attenuation correction based on the CT, an 8.4-mm gaussian filter, and 12-mm scatter filters were applied. A frame artifact in the axial view was erased from all images to improve visualization. CT was performed with 130 kVp and 25 mA.

Qualitative visual assessment of γ-camera images considered AB001 uptake in liver, kidneys, spleen, salivary glands, small bowel including contents, urinary bladder including contents, bone marrow, and metastatic lesions, in addition to blood pool. The criterion for visibility was that the structures should be clearly distinguishable from adjacent tissues (diffuse signal in an area corresponding to the organ was not sufficient). Assessment of the blood pool focused on the heart and large vessels in the mediastinum.

### Blood and Whole-Body (WB) Probe Activity Measurements

WB probe measurements and activity measurements from blood samples were acquired at 4 postinjection time points: 0–0.5, 1–2, 4–6, and 17–20 h. For patient ID03, a fifth probe measurement was obtained 2.4 h after injection.

#### WB Probe

Activity measurements were conducted with a ThermoFisher Scientific RadEye SX with an attached scintillation detector, over 60 s at a fixed distance of more than 1 m between the patient and the detector. The results were fitted with a monoexponential curve to estimate the effective WB half-life of the drug.

#### Blood Samples

One milliliter of whole blood was extracted from the blood sample, before the remaining blood was centrifuged and 1 mL of plasma was extracted. Both samples were measured on a well counter (Hidex Automatic γ-counter) with an energy window of 55–300 keV to estimate ^212^Pb activity. The samples were measured more than 12 h after blood sampling for 40 min. The samples were weighed and converted to volumes assuming densities of whole blood and plasma to be 1.06 and 1.03 g/mL, respectively (21). The activity concentrations in plasma and whole blood ($A_{\text{plasma}}$ and $A_{\text{whole}\;\text{blood}},\text{ respectively}$) were used in combination with hematocrit (HCT) to estimate the activity concentration in red blood cells ($A_{\mathrm{RBC}}$) according to

$$A_{\mathrm{RBC}}\left(T\right)=\frac{A_{\text{whole}\;\text{blood}}\left(T\right)-(1-\text{HCT})\times A_{\text{plasma}}}{\text{HCT}}.$$ Eq. 1

## RESULTS

### Patient Characteristics, Investigational Medicinal Product Administration, and Safety Assessments

Three patients were enrolled in the study between March and April 2023 and completed all study procedures. There were no screening failures. All patients had progressive mCRPC on the androgen receptor pathway inhibitor enzalutamide (Table 1).

**TABLE 1.** Patient Characteristics at Baseline

				Biomarker			
Patient	Age (y)	Time since diagnosis (y)	ECOG	PSA	ALP	Prior treatment for prostate cancer	Comorbidity
ID01	81	18	1	0.75	64	RALP, SP, HTx, BT, ARPI, EBRTp	AF, HT, HC, SN, AP, OP, HH
ID02	73	8	1	0.44	134	HTx, EBRTx, CTx, EBRTp, ARPI	CP, AMD
ID03	89	14	1	15	101	HTx, EBRTx, BT, ARPI	HT

ECOG = Eastern Cooperative Oncology Group score of performance status; PSA = prostate-specific antigen; ALP = alkaline phosphatase; RALP = robot-assisted laparoscopic prostatectomy; SP = sphincter prosthesis; HTx = antihormonal therapy; BT = bisphosphonate therapy; ARPI = androgen-receptor-pathway inhibitor; EBRTp = palliative local external-beam radiation therapy; AF = atrial fibrillation; HT = hypertension; HC = hypercholesterolemia; SN = sensory neuropathy; AP = sleep apnea; OP = osteoporosis with skeletal fractures; HH = hiatus hernia with esophagitis; EBRTx = external-beam radiation therapy for localized prostate cancer; CTx = chemotherapy; CP = columna prolapse; AMD = age-related macular degeneration.

All participants experienced at least 1 treatment-emergent adverse event (Supplemental File 1; supplemental materials are available at http://jnm.snmjournals.org). All treatment-emergent adverse events were nonserious, Common Terminology Criteria for Adverse Events grade 1 or 2, and considered unrelated to AB001.

### Imaging Results

**FIGURE 2.**
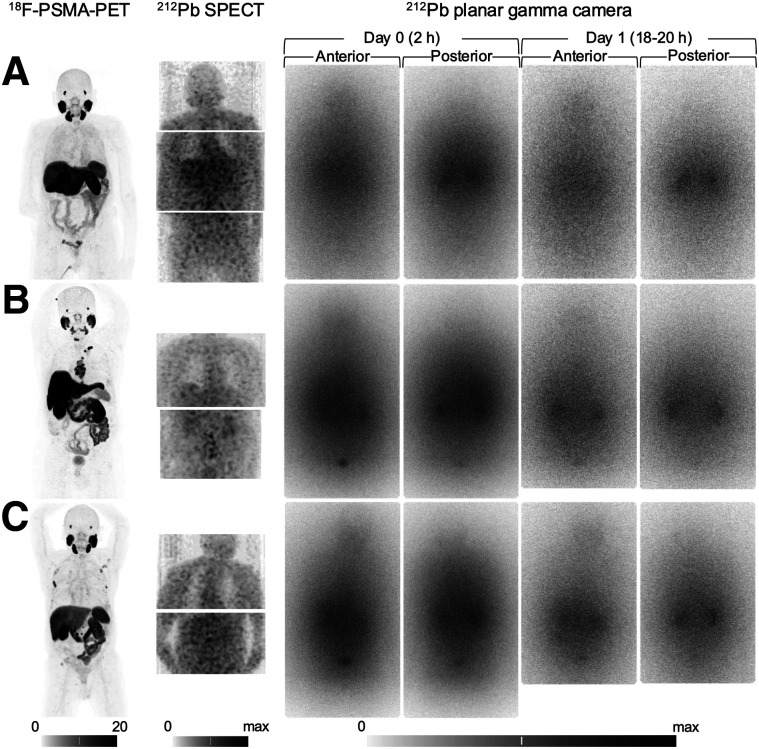
SPECT and planar γ-camera imaging of AB001: biodistribution compared with baseline ^18^F-PSMA PET for patients ID01 (A), ID02 (B), and ID03 (C). SPECT images show maximum-intensity projections with scaling of 0–maximum for each patient. PET images show maximum-intensity projections with scaling of 0–20 SUV_max_. WB γ-camera images are summed with scaling of 0–maximum.

The baseline [^18^F]F-PSMA PET/CT images confirmed patient eligibility (Fig. 2) and were used to derive SUVs for normal tissues (Supplemental File 2) and lesions.

The planar γ-camera images (Fig. 2) or SPECT/CT images allowed visualization of activity in kidneys, liver, blood pool, or urinary bladder including contents (Table 2; separate assessments for planar and SPECT images in Supplemental File 2). Some tissues were outside the SPECT field of view at 1 time point: ID01 mediastinal blood pool and salivary glands at day 1, ID02 urinary bladder including contents at day 1, ID03 urinary bladder including contents at day 0.

**TABLE 2.** Visual Assessments for Presence of ^212^Pb-Labeled PSMA-Targeting AB001 in Normal Tissues

Tissue	Patient ID01	Patient ID02	Patient ID03
Salivary glands	N	N	N
Kidneys	Y	Y	Y
Liver	N	N	Y
Spleen	N	N	N
Blood pool	Y	N	Y
Bone marrow	N	N	N
Small bowel including contents	N	N	N
Urinary bladder including contents	N*	Y	Y

* Limited bladder filling due to incontinence.

Both planar γ-camera and SPECT images were considered, and images were scored yes (Y) or no (N) if uptake was clearly visible on either.

**FIGURE 3.**
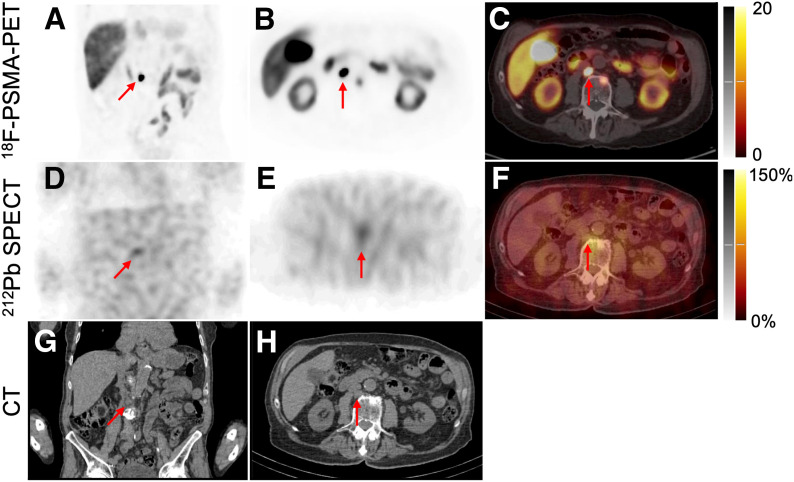
In patient ID03, retrocaval lymph node tumor metastasis (arrow) measuring 11 × 14 × 16 mm on CT (G and H), and with SUV_max_ of 77 on ^18^F-PSMA PET (A, B, and C). AB001 uptake was demonstrated by ^212^Pb SPECT imaging at 4 h after administration (D, E, and F). Lymph node tumor metastasis is seen in coronal slices (A, D, and G) and axial slices (B, C, E, F, and H). PET images are scaled at 0–20 SUV_max_, and SPECT images are scaled at 0%–150% of maximum of processed images (scale, 0–42 counts). Frame scatter artifact was removed from ^212^Pb SPECT images during postprocessing.

One retrocaval lymph node could be clearly distinguished by SPECT imaging after administration of AB001 (Fig. 3). This lesion had the highest SUV_max_, 77. The other lesions had an SUV_max_ of approximately 10–30 from the PSMA PET (Table 3).

**TABLE 3.** mCRPC Lesion Assessment by PSMA PET and Planar γ-Camera or SPECT

			PSMA PET		
Patient	Lesion location	Lesion size (cm)	SUV_max_	SUV_mean_	γ-camera imaging (AB001 uptake)
ID01	Pubic bone	NA	10.1	5.7	N
	Bladder wall	2.8 × 1.3 × 1.7	15.9	9.0	N
	Seminal vesicle	1.2 × 0.8 × 1.0	10.6	6.2	N
ID02	Sternum	NA	28.6	14.7	N
	Rib	NA	30.9	20.6	N
	Vertebra	NA	18.9	11.3	N
ID03	Lymph node, retrocaval	1.1 × 1.4 × 1.6	77.4	45.0	Y*
	Rib	NA	17.4	11.1	N
	Vertebra	NA	13.6	8.0	N

* Visualized only on SPECT/CT.

NA = not applicable.

### WB Probe Measurements and Blood Samples

**FIGURE 4.**
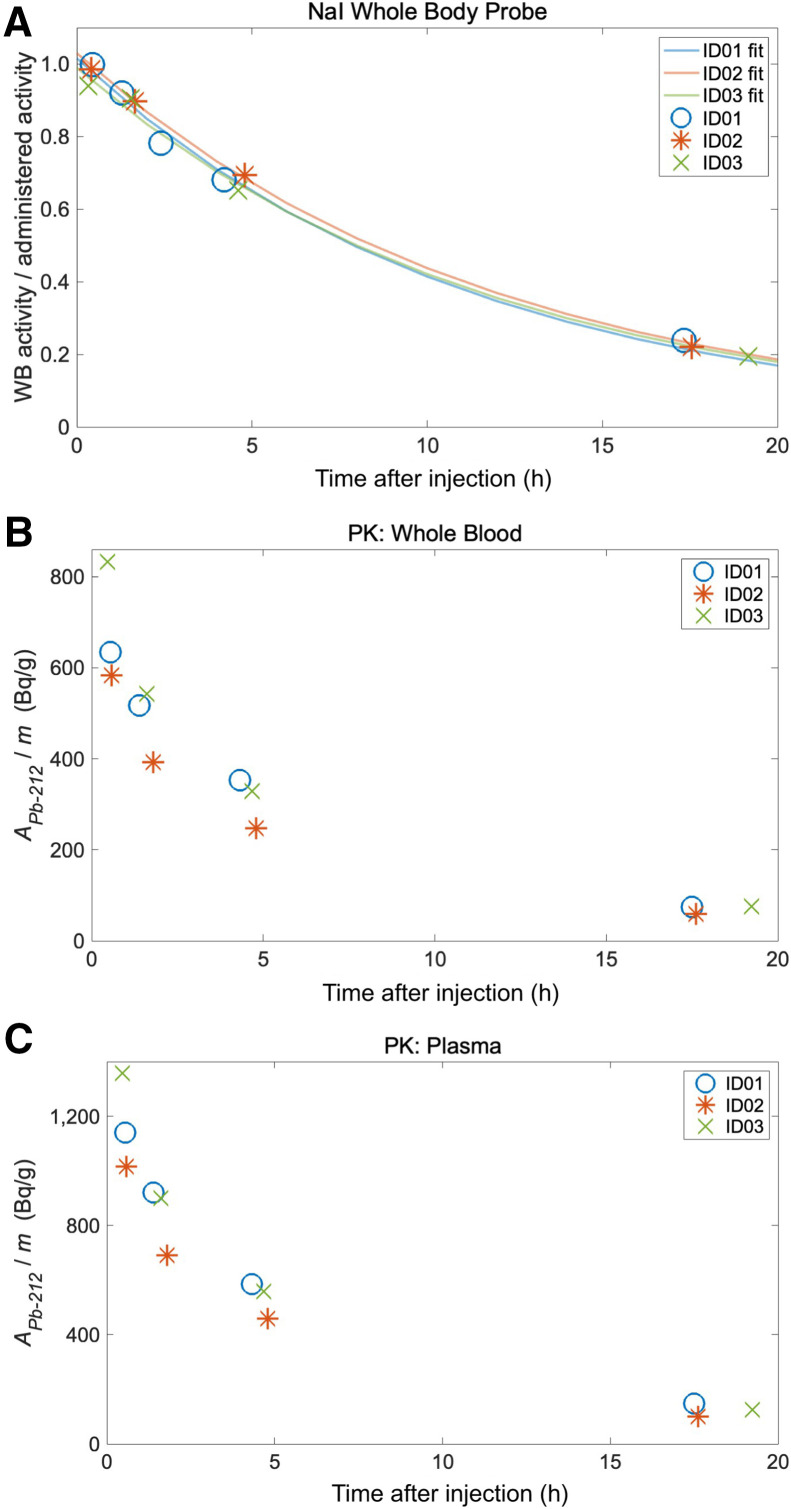
(A) WB activity as measured by NaI WB probe, after normalization against administered activity, for 3 patients. Monoexponential curve fit for each patient is included (solid lines). ^212^Pb activity pharmacokinetic (PK) measurements are shown from whole blood (B) and plasma (C).

The WB probe measurement results are presented in Figure 4. The average effective half-life to the pooled results from the 3 patients was estimated to be 8.0 h, with a 95% CI of 7.3–8.7.

The activity concentration of ^212^Pb was consistently higher in plasma than in whole blood—1.7 times higher on average, with an SD of 0.1 (Fig. 4). The activity concentrations in red blood cells calculated from Equation 1 were below zero for all but one blood sample, indicating no activity accumulation of ^212^Pb in red blood cells.

## DISCUSSION

In this phase 0 study of AB001, a ^212^Pb-PSMA–targeted radioligand, we demonstrated that γ-camera imaging was feasible even at a microdose of approximately 10 MBq. This provided a first indication of the biodistribution and normal-tissue uptake for AB001 and gave evidence of targeting of metastatic mCRPC. No complications related to administration or adverse reactions during follow-up were reported. These results warrant further clinical investigation of AB001 at therapeutic activity levels and provide important information for the design of future studies.

The γ-camera imaging performed after administration of AB001 demonstrated distinguishable uptake in kidneys and urinary bladder contents, in accordance with the expected excretion pattern from the preclinical investigations (12). Assessments of liver and blood pool were more challenging using the defined interpretation criteria; although uptake was not necessarily clearly distinguishable, the overall image patterns demonstrated diffuse activity areas indicating the presence of the radiopharmaceutical (Fig. 2; Table 2). Planar and SPECT imaging have complementary properties; hence, the normal-tissue results were presented combined. However, SPECT has an advantage when separating small lesions from surrounding tissue, as exemplified by detection of the lymph node metastasis (1.1-cm short axis) illustrated in Figure 3.

**FIGURE 5.**
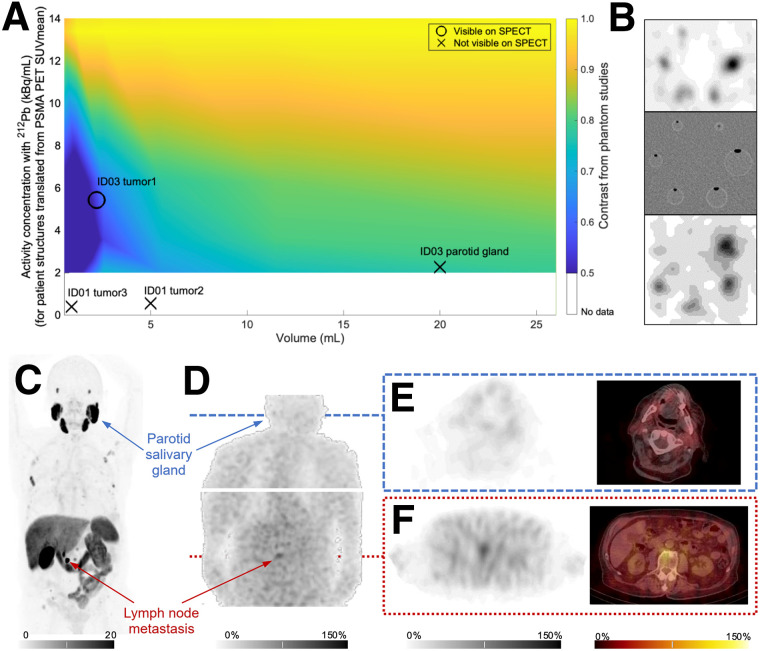
(A) Estimated activity concentrations of ^212^Pb for patient structures overlaid on phantom-derived contrast map for small volumes and low activity concentrations of ^212^Pb. Background color scale illustrates contrast as measured in spheric inserts in phantom with activity concentrations down to 2 kBq/mL, imaging ^212^Pb with SPECT reconstruction settings as in clinical study. Contrast can be seen to increase with activity concentration and volume. For patient SPECT images, some small volumes were not visualized, such as tumors of patient ID01 and salivary glands in all patients, whereas small tumor of patient ID03 was visualized. Examples of these volumes have been added to panel. Activity concentration of ^212^Pb was estimated from PSMA PET SUV_mean_, decay-corrected injected activity of ^212^Pb, and patient body weight. Although there are uncertainties in these estimates of radioligand uptake, primarily related to imaging time points and chemical differences in PET tracer and AB001, relative positions of structures should roughly reflect probability for detection. Visual assessments in combination with phantom scan results can therefore indicate that tumor–to–parotid gland uptake ratio of AB001 is higher than that of PSMA PET tracer. Methods are described in Supplemental File 3. (B) Examples of phantom data. Background color scale in (A) was constructed from these data. Top image shows central axial slice of phantom image with activity concentration of 13 kBq/mL, and bottom image is example with 2 kBq/mL. Middle image is CT of phantom setup. (C) Baseline [^18^F]F-PSMA PET/CT scan from which example structures in panel were extracted for patient ID03. (D) Background-subtracted maximum-intensity projection (anterior view) of patient ID03 from SPECT imaging after AB001, with axial sections through (E) cranium at level of parotid glands and (F) lymph node metastasis. All patient SPECT images are scaled with 0%–150% of maximum count in lymph node metastasis.

For PSMA-targeted radiotherapeutics, the kidneys, red bone marrow, salivary glands, and lacrimal glands are often considered organs at risk (9). PSMA expression in the salivary glands typically results in substantial uptake for PSMA-targeted radiopharmaceuticals. Salivary gland toxicity (xerostomia) is common for PSMA radiotherapeutics, affects patient quality of life, and may be irreversible and dose-limiting for some ^225^Ac-based therapies (22). In this study, uptake in the salivary glands was not seen. Visualization of structures may, however, be affected by the limited spatial resolution of ^212^Pb images and the low amounts of activity administered. Furthermore, factors such as geometry of structures, patient anatomy, and movement will impact the visualization probabilities. Many of these aspects are virtually unexplored in α-emitter studies, and imaging of such limited amounts of activity pushes the boundaries of what is feasible with current technology. The effects are more pronounced for smaller structures, as previously investigated in a ^212^Pb phantom study (23) and illustrated in Figure 5. However, the parotid glands are of sufficiently large volume for potential AB001 uptake to possibly be visualized. Assuming that AB001 uptake can be translated from the PSMA PET SUVs, the parotid gland of patient ID03 can, for instance, be estimated to have an uptake of 2.3 kBq/mL (corresponding to a PSMA PET SUV_mean_ of 19.8 for the 62-kg patient and a decay-corrected injected activity of 9 MBq of ^212^Pb). Assuming equal parotid uptake for AB001 and ^18^F-PSMA-1007, the expected SPECT image contrast of the gland would be slightly higher than that of the small visible lymph node lesion (Fig. 5). This suggests that parotid salivary gland uptake may be lower for AB001 than for ^18^F-PSMA-1007. AB001 has the same PSMA-binding moiety as PSMA-1007 and PSMA-617 but a differentiated linker and chelator (11). These features of ligand design should result in a slightly more hydrophobic compound than PSMA-617, which may reduce and slow the rate of nonspecific uptake of AB001 into the salivary glands, potentially contributing to a lack of uptake observed on the images.

Estimation of absorbed dose to normal organs and tumors was outside the scope of this ^212^Pb microdose feasibility study, which assessed only 2 imaging time points. For future characterization of AB001, γ-camera imaging could be considered 2–3, 5, 12, 24, and 40 h after injection, corresponding to ⅓, ⅔, 1½, 3, and 5 times the effective 8-h half-life of the later clearance phase (24). If the therapy is administered in the morning, the first 2 imaging time points are easily feasible in a trial setting. However, the 12- and 40-h time points may be more practically challenging. The first of these two may be moved earlier the same day, and the second to the end of day 1 or the start of day 2. Furthermore, for dosimetry based on ^212^Pb SPECT images, there will be a trade-off between quantification accuracy and characterizing latter parts of the time–activity curves. Hence, the optimal imaging schedule will also depend on the administered activity. If 100 MBq are administered, approximately 3 MBq would remain in the patient at 40 h after injection. Accounting for some interpatient variation, this may be pushing into the lower end of what can be quantified without risking overestimation (23), supporting an earlier final imaging time point. If 200 MBq or more are administered, images acquired 48 h after administration could potentially be quantified for dosimetry. To support image-based dosimetry, blood samples should be collected in future trials, also to investigate pharmacokinetic modeling as a basis for dosimetry. More blood sampling time points should then be included, especially to better characterize the latter part of the curve. Urine measurements could also be considered both to support the WB clearance measurements and to validate modeling and imaging results (4).

In this study, only 1 metastatic lesion was visualized with AB001 uptake on postinjection SPECT/CT. Uptake of AB001 was not demonstrated for the other metastatic lesions. This observation may be attributed to the lower PSMA expression of these metastases due to tumor biology and limited size of metastases, combined with the microdose of AB001 administered and imaging system limitations. For example, an SUV_mean_ of 6.2 for the seminal vesicle lesion in patient ID01 corresponds to 0.6 kBq of ^212^Pb per milliliter. The possible difference in expected visibility for such a lesion, with a volume of 0.9 cm^3^, versus the visible lesion in patient ID03 with a volume of approximately 2.2 cm^3^ and an uptake level estimated to be 5.4 kBq/mL, is apparent (Fig. 5). Although we believe the inclusion and exclusion criteria would not diminish the generalizability of the drug for an mCRPC population, the subtherapeutic dose of AB001 appropriate for a phase 0 imaging study made patient identification within a short recruitment period challenging. Patients with rapidly progressing extensive mCRPC were not recruited and instead were considered for ^177^Lu-PSMA–targeted treatment. The 3 patients who were included had progressive mCRPC that did not require urgent radioligand treatment and had smaller lesions with lower PSMA expression. These factors added further complexity to the considerable challenges of lesion visualization.

AB001 was safely administered, with no related adverse reactions observed during injection or follow-up. The evaluation of activity in whole blood, plasma, and red blood cells indicated very low to no activity concentration in red blood cells. As free ^212^Pb in blood is subjected to bind to red blood cells (25), this implies a very limited amount of ^212^Pb released from the chelator. Although this should be further validated, the results indicate that AB001 is a stable radioligand in vivo. The results also suggest a clearance roughly similar to that for other PSMA-targeted radioligand therapies (26). Similar renal kinetics could, in theory, imply a relatively higher kidney-absorbed dose due to the shorter half-life of the radionuclide ^212^Pb (27). However, nonclinical biodistribution studies measuring activity in normal tissues have characterized AB001 and included a direct comparison with ^212^Pb-PSMA-617 at 2 h after injection, indicating lower kidney uptake for AB001 (11,12). Furthermore, factors such as the short range of the emitted α-particles may also reduce the actual kidney toxicity, and other tissues may turn out to be limiting for α-emitter based treatments. If salivary gland uptake of AB001 is relatively slow, this would fit nicely with the 10.6-h half-life of ^212^Pb and might contribute to beneficial kinetics, potentially reducing the salivary gland toxicity commonly seen in PSMA-targeted radioligand therapies. To address these points, AB001 needs to be investigated in further clinical trials including both thorough safety evaluations and dosimetry.

As expected for a subtherapeutic 10-MBq administration of AB001, there was no observed therapeutic effect by prostate-specific antigen or alkaline phosphatase biomarker analysis. Estimating the activity amounts of AB001 required for therapeutic benefit is challenging, particularly because of the limited clinical data available for ^212^Pb. Early clinical data on the therapeutic effect of ^212^Pb-radioligands have been published only for a different indication, SSTR2 analogs for treatment of neuroendocrine tumors, which precludes direct comparison of activity levels. Phase 1 and 2 studies of ^212^Pb-DOTAMTATE demonstrated a 57% objective response rate in 44 patients treated at 2.50 MBq/kg (i.e., equivalent to 175 MBq for a 70-kg patient) administered 4 times in 8-wk cycles (28,29). For mCRPC, to date only α-radioligand therapies using ^225^Ac have been extensively explored (30); however, comparing dosage requirements between different nuclides is also not straightforward. Early studies with ^225^Ac-PSMA-617 or ^225^Ac-PSMA-I&T have reported an administered activity per cycle ranging between 1.5 and 13 MBq (30), but no optimized therapeutic activity has yet been established. The total α- and β-energy emitted by ^225^Ac is 3.2 times higher than for ^212^Pb, and the half-life of ^225^Ac is much longer (10 d, compared with 10.6 h for ^212^Pb). A simple estimate, based only on monoexponential WB clearance and not organ uptake, and assuming the same biologic half-lives, gives absorbed doses received with ^225^Ac-PSMA 11.2 times higher per administered activity than for a ^212^Pb-PSMA treatment. Hence, to test activity levels compared with ^225^Ac-PSMA studies, one might start with activities of approximately 100 MBq AB001 per administration over multiple cycles. Future studies should aim to optimize the dosage of AB001 not only by exploring the optimal activity to administer per cycle but also by investigating the optimal frequency of dosing given the relatively fast clearance of AB001 due to the short half-life of ^212^Pb.

## CONCLUSION

The ^212^Pb-PSMA–targeted radioligand AB001 was safely administered to mCRPC patients in this phase 0 study. γ-camera imaging was feasible but challenging at a microdose, and care should be exercised in visual assessments of smaller structures. AB001 demonstrated metastatic targeting, albeit for only 1 lesion in this limited population. The biodistribution was promising, including potentially low salivary gland uptake that warrants further exploration. AB001 constitutes a ^212^Pb-based treatment that merits investigation in a therapeutic phase 1 clinical study.

## DISCLOSURE

The clinical trial was sponsored and funded by ARTBIO. Monika Kvassheim is financially supported by the South-Eastern Norway Regional Health Authority (project 2020028, Oslo, Norway). Øyvind Bruland and Roy Larsen are scientific cofounders and indirect shareholders in ARTBIO and, respectively, are on the clinical scientific advisory board and on the board of directors in ARTBIO. Lily Bouzelmat and Vicki Jardine are employees and holders of options in ARTBIO. No other potential conflict of interest relevant to this article was reported.

## Notes

KEY POINTS **QUESTION:** Is γ-gamma camera imaging of ^212^Pb-based PSMA radioligands for mCRPC patients feasible? **PERTINENT FINDINGS:** Imaging of a microdose 10 MBq of the ^212^Pb-based PSMA-targeted radioligand AB001 was challenging but feasible. Uptake was demonstrated in one 11-mm metastatic abdominal lymph node lesion. The salivary glands were not visualized, indicating relatively low uptake. **IMPLICATIONS FOR PATIENT CARE:** AB001, a ^212^Pb-based PSMA-targeted α-alpha radioligand therapy, should be investigated in a therapeutic dose-finding clinical study.

## References

[1] Hawkey NM Sartor AO Morris MJ Armstrong AJ. Prostate-specific membrane antigen-targeted theranostics: past, present, and future approaches. Clin Adv Hematol Oncol. 2022;20:227–238. 35389387 PMC9423035

[2] Sartor O de Bono J Chi KN.; VISION Investigators. Lutetium-177-PSMA-617 for metastatic castration-resistant prostate cancer. N Engl J Med. 2021;385:1091–1103. 34161051 10.1056/NEJMoa2107322 PMC8446332

[3] Sgouros G Bodei L McDevitt MR Nedrow JR. Radiopharmaceutical therapy in cancer: clinical advances and challenges. Nat Rev Drug Discov. 2020;19:589–608. 32728208 10.1038/s41573-020-0073-9 PMC7390460

[4] Stokke C Gnesin S Tran-Gia J. EANM guidance document: dosimetry for first-in-human studies and early phase clinical trials. Eur J Nucl Med Mol Imaging. 2024;51:1268–1286. 38366197 10.1007/s00259-024-06640-x PMC10957710

[5] Satapathy S Sood A Das CK Mittal BR. Evolving role of ^225^Ac-PSMA radioligand therapy in metastatic castration-resistant prostate cancer: a systematic review and meta-analysis. Prostate Cancer Prostatic Dis. 2021;24:880–890. 33746213 10.1038/s41391-021-00349-w

[6] Yadav MP Ballal S Bal C. Efficacy and safety of ^177^Lu-PSMA-617 radioligand therapy in metastatic castration-resistant prostate cancer patients. Clin Nucl Med. 2020;45:19–31. 31789908 10.1097/RLU.0000000000002833

[7] Feuerecker B Tauber R Knorr K. Activity and adverse events of actinium-225-PSMA-617 in advanced metastatic castration-resistant prostate cancer after failure of lutetium-177-PSMA. Eur Urol. 2021;79:343–350. 33293081 10.1016/j.eururo.2020.11.013

[8] Ballal S Yadav MP Sahoo RK Tripathi M Dwivedi SN Bal C. ^225^Ac-PSMA-617-targeted alpha therapy for the treatment of metastatic castration-resistant prostate cancer: a systematic review and meta-analysis. Prostate. 2021;81:580–591. 33905559 10.1002/pros.24137

[9] Sathekge MM Bruchertseifer F Vorster M Morgenstern A Lawal IO. Global experience with PSMA-based alpha therapy in prostate cancer. Eur J Nucl Med Mol Imaging. 2021;49:30–46. 34173838 10.1007/s00259-021-05434-9 PMC8712297

[10] Zimmermann R. Is ^212^Pb Really Happening? The post-^177^Lu/^225^Ac blockbuster? J Nucl Med. 2024;65:176–177. 38176723 10.2967/jnumed.123.266774

[11] Stenberg VY Juzeniene A Chen Q Yang X Bruland OS Larsen RH. Preparation of the alpha-emitting prostate-specific membrane antigen targeted radioligand [^212^Pb]Pb-NG001 for prostate cancer. J Labelled Comp Radiopharm. 2020;63:129–143. 31919866 10.1002/jlcr.3825

[12] Stenberg VY Larsen RH Ma LW. Evaluation of the PSMA-binding ligand ^212^Pb-NG001 in multicellular tumour spheroid and mouse models of prostate cancer. Int J Mol Sci. 2021;22:4815. 10.3390/ijms22094815 PMC8124365 34062920

[13] Mikalsen LTG Kvassheim M Stokke C. Optimized SPECT Imaging of ^224^Ra alpha-particle therapy by ^212^Pb photon emissions. J Nucl Med. 2023;64:1131–1137. 37268424 10.2967/jnumed.122.264455 PMC10315694

[14] Auranen K Mccutchan EA. Nuclear data sheets for A=212. NuDat 3.0 website. https://www.nndc.bnl.gov/nudat/. Published 2020. Accessed March 5, 2025.

[15] Meredith RF Torgue J Azure MT. Pharmacokinetics and imaging of ^212^Pb-TCMC-trastuzumab after intraperitoneal administration in ovarian cancer patients. Cancer Biother Radiopharm. 2014;29:12–17. 24229395 10.1089/cbr.2013.1531 PMC3869429

[16] Michler E Kastner D Brogsitter C. First-in-human SPECT/CT imaging of [^212^Pb]Pb-VMT-alpha-NET in a patient with metastatic neuroendocrine tumor. Eur J Nucl Med Mol Imaging. 2024;51:1490–1492. 37991526 10.1007/s00259-023-06529-1 PMC10957691

[17] Griffiths MR Pattison DA Latter M. First-in-human ^212^Pb-PSMA-targeted α-therapy SPECT/CT imaging in a patient with metastatic castration-resistant prostate cancer. J Nucl Med. 2024;65:664. 38423783 10.2967/jnumed.123.267189 PMC10995529

[18] Oken MM Creech RH Tormey DC. Toxicity and response criteria of the Eastern Cooperative Oncology Group. Am J Clin Oncol. 1982;5:649–655. 7165009

[19] Li RG Stenberg VY Larsen RH. An experimental generator for production of high-purity ^212^Pb for use in radiopharmaceuticals. J Nucl Med. 2023;64:173–176. 35798556 10.2967/jnumed.122.264009 PMC9841245

[20] Giesel FL Hadaschik B Cardinale J. F-18 labelled PSMA-1007: biodistribution, radiation dosimetry and histopathological validation of tumor lesions in prostate cancer patients. Eur J Nucl Med Mol Imaging. 2017;44:678–688. 27889802 10.1007/s00259-016-3573-4 PMC5323462

[21] Phillips RA Vanslyke DD Hamilton PB Dole VP Emerson K Archibald RM. Measurement of specific gravities of whole blood and plasma by standard copper sulfate solutions. J Biol Chem. 1950;183:305–330.

[22] Kratochwil C Bruchertseifer F Rathke H. Targeted alpha-therapy of metastatic castration-resistant prostate cancer with ^225^Ac-PSMA-617: dosimetry estimate and empiric dose finding. J Nucl Med. 2017;58:1624–1631. 28408529 10.2967/jnumed.117.191395

[23] Kvassheim M Revheim MR Stokke C. Quantitative SPECT/CT imaging of lead-212: a phantom study. EJNMMI Phys. 2022;9:52. 35925521 10.1186/s40658-022-00481-z PMC9352840

[24] Kearfott KJ. ICRU report 67: absorbed-dose specification in nuclear medicine. Health Phys. 2003;85:113–113.

[25] Occupational intakes of radionuclides: part 3. ICRP publication 137. Ann ICRP. 2017;46:1–486. 10.1177/0146645317734963 29380630

[26] Kurth J Krause BJ Schwarzenböck SM Stegger L Schäfers M Rahbar K. External radiation exposure, excretion, and effective half-life in ^177^Lu-PSMA-targeted therapies. EJNMMI Res. 2018;8:32. 29651569 10.1186/s13550-018-0386-4 PMC5897276

[27] Stokke C Kvassheim M Blakkisrud J. Radionuclides for targeted therapy: physical properties. Molecules. 2022;27:5429. 10.3390/molecules27175429 PMC9457625 36080198

[28] Delpassand ES Tworowska I Esfandiari R. Targeted alpha-emitter therapy with ^212^Pb-DOTAMTATE for the treatment of metastatic SSTR-expressing neuroendocrine tumors: first-in-humans dose-escalation clinical trial. J Nucl Med. 2022;63:1326–1333. 34992153 10.2967/jnumed.121.263230 PMC9454455

[29] Strosberg J Naqvi S Cohn AL. Safety, tolerability and efficacy of ^212^Pb-DOTAMTATE as a targeted alpha therapy for subjects with unresectable or metastatic somatostatin receptor-expressing gastroenteropancreatic neuroendocrine tumors (SSTR+ GEP-NETs): a phase 2 study. J Clin Oncol. 2024;42:4020–4020.

[30] Lee DY Kim Y-I. Effects of ^225^Ac-labeled prostate-specific membrane antigen radioligand therapy in metastatic castration-resistant prostate cancer: a meta-analysis. J Nucl Med. 2022;63:840–846. 34503960 10.2967/jnumed.121.262017

